# Hepatocyte Growth Factor in Synaptic Plasticity and Alzheimer's Disease

**DOI:** 10.1100/tsw.2010.49

**Published:** 2010-03-16

**Authors:** Shiv K. Sharma

**Affiliations:** National Brain Research Centre, Manesar, Haryana-122050, India

**Keywords:** hepatocyte growth factor, HGF, HGF receptor, long-term potentiation, Alzheimer’s disease

## Abstract

The hepatocyte growth factor (HGF) was initially identified as a protein that promoted growth of hepatocytes. It regulates proliferation and survival of different types of cells. HGF signaling, which is initiated by its binding to a receptor tyrosine kinase, plays critical roles during development. HGF and its receptor are also present in brain cells. This review describes the role of HGF in hippocampal neurons, synaptic plasticity, and the memory impairment condition, Alzheimer's disease.

## INTRODUCTION

Hepatocyte growth factor (HGF), a heparin-binding protein, was identified as a potent mitogen for hepatocytes[1]. It is made up of disulfide bond–linked light and heavy chains. Molecular cloning has revealed that the heavy and light chains are encoded by the same mRNA[2,3]. HGF is thus synthesized as a single-chain protein, which is then proteolytically processed to generate heavy and light chains[4,5].

HGF signaling starts with its binding to the high-affinity tyrosine kinase receptor, Met. Met is made up of disulfide bond–linked alpha and beta chains. The alpha chain is extracellular, whereas the beta chain is a transmembrane peptide. Upon HGF binding, the Met receptor undergoes dimerization and phosphorylation. When phosphorylated, the tyrosine residues in the beta subunit serve as docking sites for the downstream signaling mediators and lead to activation of signaling systems, including the extracellular signal-regulated kinase (ERK) and the phosphatidylinositol-3-kinase (PI3K) pathways[6,7]. The HGF-Met signaling is regulated by the HGF activator (HGFA) and its inhibitor (HGFAI). HGFA is a protease that acts on the precursor protein and produces active HGF. HGFAI blocks the activation of HGFA[8]. HGF signaling is important during development since genetic manipulation of this signaling leads to embryonic lethality. HGF is also a survival factor for many cell types[7].

In addition to other cells of the body, HGF and its receptor are also expressed in the brain, including the hippocampal region[9,10,11]. Furthermore, HGF activates Met and the downstream component, ras proteins, in the neurons[12], indicating the presence of a functional HGF signaling system. The following sections describe the role of HGF in hippocampal neurons, long-term potentiation, and Alzheimer's disease.

## HGF AND HIPPOCAMPAL NEURONS

Tyndall and Walikonis[13] examined activity-dependent regulation of HGF signaling in hippocampal neurons. They found that treatment of the hippocampal neurons with glutamate, an excitatory neurotransmitter, increased HGF staining on the neurons. In addition, there was an increase in HGF levels in the culture media after glutamate treatment. Importantly, glutamate treatment led to enhanced phosphorylation of Met. These investigators then used bicuculline to increase synaptic activity and examined the effects on Met activation. Bicuculline is an antagonist of the gamma-aminobutyric acid (GABA) receptor and thus would block the inhibitory input to the neurons, leading to an increase in synaptic activity. Similar to glutamate, bicuculline application also led to Met activation. Moreover, the pattern of Met activation with bicuculline was similar to that with the glutamate. These results show that synaptic activity modulates HGF signaling in hippocampal neurons.

Excitatory synaptic transmission plays crucial roles in maintaining proper physiology of the nervous system and in experience-dependent plasticity. Tyndall and Walikonis[14] found that the HGF receptor was present at the excitatory synapses in hippocampal neurons. More importantly, Met was clustered in the postsynaptic density region of the synapse. In addition, HGF enhanced the expression of glutamate receptor subunits. Furthermore, HGF increased the number of PSD95 and synapsin clusters, and enhanced KCl depolarization-induced glutamate release from the cultured hippocampal cells[15]. Activity-dependent dendritic remodeling is a prominent feature during development and also in the mature nervous system. HGF plays crucial roles in dendritic arborization[16,17]. Walikonis and colleagues found that HGF enhanced dendritic arborization in the hippocampal neurons and this effect required N-methyl-d-aspartate (NMDA) receptor activity[17]. Moreover, HGF treatment increased $Ca^{++}$ levels in the cells in a NMDA receptor activity-dependent manner. Lim and Walikonis[18] further showed that HGF-induced dendritic growth required Akt activity. Collectively, these results show that HGF modulates synaptic function and enhances dendritic arborization.

## EFFECTS OF HGF ON LONG-TERM POTENTIATION

Activity-dependent plasticity is important for proper development of the nervous system. It is also critical for memory formation. Long-term potentiation (LTP) refers to the phenomenon of an increase in synaptic strength. Since its discovery by Bliss and Lomo[19] in the hippocampus, it is widely studied as a candidate for cellular mechanism of memory formation[20,21]. LTP can be induced in a variety of pathways in the brain, and can last for hours *in vitro* and for a much longer time *in vivo*. The mechanisms of LTP vary depending on the neuronal pathway and the induction protocol. LTP at the CA3-CA1 synapses in the hippocampus has been widely used for the studies of mechanisms and properties of LTP. In this pathway, the NMDA receptor, a type of glutamate receptor, plays critical roles in the induction of LTP. Several signaling molecules, including calmodulin-dependent kinase, protein kinase A, protein kinase C, and ERK, are important for LTP[22,23]. In addition, brain-derived growth factor (BDNF) plays important roles in LTP[24]. Similar to memory, LTP also has different phases that can be distinguished, based on their molecular requirement. For example, the late phase of LTP requires changes in protein and RNA synthesis, whereas the early phase of LTP is independent of transcription and translation.

Akimoto and colleagues[11] studied the role of HGF in the formation of LTP in the hippocampus. They found that exogenously applied HGF increased the magnitude of LTP at the CA3-CA1 synapses. Importantly, HGF had no effects on basal synaptic transmission. In addition, paired pulse facilitation, a short-term plasticity, was also unaffected by HGF. How does HGF enhance LTP? Since the activation of alpha-amino-3-hydroxy-5-methyl-4-isoxazole propionic acid (AMPA) and NMDA receptors is known to play critical roles in LTP, the authors examined whether HGF affected AMPA or NMDA receptor activity. They found that HGF increased the NMDA-induced current, but had no effects on AMPA-induced current in the hippocampal neurons. These results suggest that the effect of HGF on the NMDA receptor current could contribute to its enhancing effect on LTP. Interestingly, HGF did not affect longterm depression, another kind of synaptic plasticity in which there is a decrease in synaptic strength. It remains to be examined whether HGF-induced enhancement of LTP follows the mechanisms that are important during normal LTP formation. For example, does HGF-induced LTP enhancement require activation of ERK, which has been shown to play crucial roles in synaptic plasticity and memory in different model systems[23,25]? Also, does HGF-facilitated LTP require new protein and RNA synthesis? In addition, it would be interesting to examine whether blocking HGF signaling affects normally induced LTP.

## HGF AND ALZHEIMER'S DISEASE

Alzheimer's disease (AD) is a progressive, neurodegenerative disorder and is the most common form of dementia in the elderly. Since its discovery by Alois Alzheimer more than a century ago, tremendous efforts have been directed towards identifying the molecular mechanisms involved in AD. Pathologically, AD is characterized by the presence of amyloid plaques and neurofibrillary tangles in the brain. It is widely accepted that amyloid beta (A-beta), which is generated by proteolytic processing of amyloid precursor protein, plays important roles in the development of AD. A-beta causes neurodegeneration and neuroinflammation, and impairs LTP and memory.

Considerable efforts are directed towards identifying agents that can protect neurons, reduce inflammation, and improve memory. HGF shows beneficial effects in the amyotrophic lateral sclerosis (ALS) model[26] and attenuates memory deficits after cerebral ischemia[27]. In addition to neurons, HGF is present in other cells of the nervous system, such as astrocytes, and its levels are increased in AD patients[28,29]. Furthermore, HGF levels are increased in the cerebrospinal fluid of AD patients, perhaps due to white matter damage[30]. Takeuchi et al. recently explored whether the cognitive deficits in an animal model of AD can be ameliorated using the HGF gene therapy approach[31]. They found that ultrasound-mediated transfer of human HGF plasmid DNA into mouse brain resulted in the expression of human HGF. In addition, there was an increase in the expression of endogeneous mouse HGF. Furthermore, the HGF receptor was also up-regulated after HGF gene transfer, suggesting an enhancement in HGF signaling. Having established that transfer of HGF gene in the brain leads to its synthesis, the authors then examined whether enhancing the levels of HGF had any effects on cognitive ability. To create an AD model, they injected A-beta peptide into the mouse brain using an intracerebroventricular route. The injection of A-beta peptide recapitulates some of the pathological features of AD and has been used in several studies to examine different aspects of AD. Injection of A-beta peptide impaired cognitive ability. However, HGF expression led to improvement in cognitive performance in the A-beta–injected animals.

How does HGF improve the performance of animals in cognitive tasks? The authors noticed several beneficial effects after HGF expression that could contribute to improvement in cognitive ability. They found a decrease in the blood-vessel density after injection of A-beta peptide that improved after HGF expression. The increase in blood-vessel density after HGF expression is in agreement with the known angiogenic property of HGF. The results suggest that increased blood-vessel density could, at least in part, contribute to improvement in cognitive tasks. A decrease in cerebral blood flow is observed in AD patients[32].

Oxidative stress has been implicated in AD[33]. Does expression of HGF in the mouse model of AD have any effects on oxidative stress? Takeuchi et al.[31] found that A-beta injection induced oxidative stress in the hippocampus of the animals. However, HGF expression showed beneficial effects in decreasing oxidative stress. In this regard, the action of HGF is similar to the actions of antioxidants that have been shown to have beneficial effects in the experimental setup. The authors further found an increase in the expression of BDNF, both at the mRNA and protein levels, after HGF expression. Thus, HGF expression improves memory deficits in the A-beta–injected animals by a combination of its effects on blood-vessel density, oxidative stress, and BDNF expression.

## CONCLUSIONS

HGF increases dendritic arborization and enhances LTP in the hippocampus. The fact that A-beta impairs LTP[34] and that HGF enhances LTP raises the possibility that HGF treatment could be beneficial in overcoming synaptic plasticity deficits induced by A-beta. As described earlier, HGF activates ERK signaling. Since ERK plays important roles in synaptic plasticity and memory, it would be interesting to examine whether A-beta affects HGF-induced ERK signaling. Growth factors offer a promising approach for the treatment of neurodegenerative diseases. However, therapeutic use of growth factors is limited due to their inability to permeate the blood-brain barrier (BBB). The development of small molecules that can cross the BBB and increase the production of endogenous growth factors is in progress. Recently, S18986, a modulator of AMPA receptors was shown to increase the levels of BDNF under certain conditions[35,36]. It remains to be examined whether AMPA receptor modulators affect the levels of HGF.

## ACKNOWLEDGMENTS

I thank Preeti Yadav for her help in the preparation of this manuscript. This work was supported by a grant from the Council of Scientific and Industrial Research, India, and a core grant to the National Brain Research Centre from the Department of Biotechnology, India.

## References

1. Nakamura, T., Nawa, K., and Ichihara, A. (1984) Partial purification and characterization of hepatocyte growth factor from serum of hepatectomized rats. *Biochem. Biophys. Res. Commun.* **122,** 1450–1459.
2. Miyazawa, K., Tsubouchi, H., Naka, D., Takahashi, K., Okigaki, M., Arakaki, N., Nakayama, H., Hirono, S., Sakiyama, O., Takahashi, K., et al. (1989) Molecular cloning and sequence analysis of cDNA for human hepatocyte growth factor. *Biochem. Biophys. Res. Commun.* **163,** 967–973.
3. Nakamura, T., Nishizawa, T., Hagiya, M., Seki, T., Shimonishi, M., Sugimura, A., Tashiro, K., and Shimizu, S. (1989) Molecular cloning and expression of human hepatocyte growth factor. *Nature* **342,** 440–443.
4. Naka, D., Ishii, T., Yoshiyama, Y., Miyazawa, K., Hara, H., Hishida, T., and Kidamura, N. (1992) Activation of hepatocyte growth factor by proteolytic conversion of a single chain form to a heterodimer. *J. Biol. Chem*. **267,** 20114–20119.
5. Naldini, L., Tamagnone, L., Vigna, E., Sachs, M., Hartmann, G., Birchmeier, W., Daikuhara, Y., Tsubouchi, H., Blasi, F., and Comoglio, P.M. (1992) Extracellular proteolytic cleavage by urokinase is required for activation of hepatocyte growth factor/scatter factor. *EMBO J.* **11,** 4825–4833.
6. Rosário, M. and Birchmeier, W. (2003) How to make tubes: signaling by the Met receptor tyrosine kinase. *Trends Cell Biol*. **13,** 328–335.
7. Tulasne, D. and Foveau, B. (2008) The shadow of death on the MET tyrosine kinase receptor. *Cell Death Differ*. **15,** 427–434.
8. Zachow, R. and Uzumcu, M. (2007) The hepatocyte growth factor system as a regulator of female and male gonadal function. *J. Endocrinol*. **195,** 359–371.
9. Jung, W., Castren, E., Odenthal, M., Vande Woude, G.F., Ishii, T., Dienes, H.P., Lindholm, D., and Schirmacher, P. (1994) Expression and functional interaction of hepatocyte growth factor-scatter factor and its receptor c-met in mammalian brain. *J. Cell Biol*. **126,** 485–494.
10. Honda, S., Kagoshima, M., Wanaka, A., Tohyama, M., Matsumoto, K., and Nakamura, T. (1995) Localization and functional coupling of HGF and c-Met/HGF receptor in rat brain: implication as neurotrophic factor. *Brain Res. Mol. Brain Res*. **32,** 197–210.
11. Akimoto, M., Baba, A., Ikeda-Matsuo, Y., Yamada, M.K., Itamura, R., Nishiyama, N., Ikegaya, Y., and Matsuki, N. (2004) Hepatocyte growth factor as an enhancer of nmda currents and synaptic plasticity in the hippocampus. *Neuroscience* **128,** 155–162.
12. Yamagata, T., Muroya, K., Mukasa, T., Igarashi, H., Momoi, M., Tsukahara, T., Arahata, K., Kumagai, H., and Momoi, T. (1995) Hepatocyte growth factor specifically expressed in microglia activated Ras in the neurons, similar to the action of neurotrophic factors. *Biochem. Biophys. Res. Commun*. **210,** 231–237.
13. Tyndall, S.J. and Walikonis, R.S. (2007) Signaling by hepatocyte growth factor in neurons is induced by pharmacological stimulation of synaptic activity. *Synapse* **61,** 199–204.

14. Tyndall, S.J. and Walikonis, R.S. (2006) The receptor tyrosine kinase Met and its ligand hepatocyte growth factor are clustered at excitatory synapses and can enhance clustering of synaptic proteins. *Cell Cycle* **5,** 1560–1568.
15. Nakano, M., Takagi, N., Takagi, K., Funakoshi, H., Matsumoto, K., Nakamura, T., and Takeo, S. (2007) Hepatocyte growth factor promotes the number of PSD-95 clusters in young hippocampal neurons. *Exp. Neurol*. **207,** 195–202.
16. Gutierrez, H., Dolcet, X., Tolcos, M., and Davies, A. (2004) HGF regulates the development of cortical pyramidal dendrites. *Development* **131,** 3717–3726.
17. Tyndall, S.J., Patel, S.J., and Walikonis, R.S. (2007) Hepatocyte growth factor-induced enhancement of dendritic branching is blocked by inhibitors of N-methyl-D-aspartate receptors and calcium/calmodulin-dependent kinases. *J. Neurosci. Res*. **85,** 2343–2351.
18. Lim, C.S. and Walikonis, R.S. (2008) Hepatocyte growth factor and c-Met promote dendritic maturation during hippocampal neuron differentiation via the Akt pathway. *Cell. Signal*. **20,** 825–835.
19. Bliss, T.V. and Lomo, T. (1973) Long-lasting potentiation of synaptic transmission in the dentate area of the anaesthetized rabbit following stimulation of the perforant path. *J. Physiol*. **232,** 331–356.
20. Bliss, T.V. and Collingridge, G.L. (1993) A synaptic model of memory: long-term potentiation in the hippocampus. *Nature* **361,** 31–39.
21. Lynch, M.A. (2004) Long-term potentiation and memory. *Physiol. Rev.* **84,** 87–136.
22. Roberson, E.D., English, J.D., and Sweatt, J.D. (1996) A biochemist's view of long-term potentiation. *Learn. Mem*. **3,** 1–24.
23. Sweatt, J.D. (2001) The neuronal MAP kinase cascade: a biochemical signal integration system subserving synaptic plasticity and memory. *J. Neurochem*. **76,** 1–10.
24. Lu, Y., Christian, K., and Lu, B. (2008) BDNF: a key regulator for protein synthesis-dependent LTP and long-term memory? *Neurobiol. Learn. Mem*. **89,** 312–323.
25. Sharma, S.K. and Carew, T.J. (2004). The roles of MAPK cascades in synaptic plasticity and memory in Aplysia: facilitatory effects and inhibitory constraints. *Learn. Mem*. **11,** 373–378.
26. Sun, W., Funakoshi, H., and Nakamura, T. (2002) Overexpression of HGF retards disease progression and prolongs life span in a transgenic mouse model of ALS. *J. Neurosci*. **22,** 6537–6548.
27. Date, I., Takagi, N., Takagi, K., Kago, T., Matsumoto, K., Nakamura, T., and Takeo, S. (2004) Hepatocyte growth factor attenuates cerebral ischemia-induced learning dysfunction. *Biochem. Biophys. Res. Commun*. **319,** 1152–1158.
28. Yamada, T., Tsubouchi, H., Daikuhara, Y., Prat, M., Comoglio, P.M., McGeer, P.L., and McGeer, E.G. (1994) Immunohistochemistry with antibodies to hepatocyte growth factor and its receptor protein (c-MET) in human brain tissues. *Brain Res*. **637,** 308–312.
29. Fenton, H., Finch, P.W., Rubin, J.S., Rosenberg, J.M., Taylor, W.G., Kuo-Leblanc, V., Rodriguez-Wolf, M., Baird, A., Schipper, H.M., and Stopa, E.G. (1998) Hepatocyte growth factor (HGF/SF) in Alzheimer's disease. *Brain Res*. **779,** 262–270.
30. Tsuboi, Y., Kakimoto, K., Nakajima, M., Akatsu, H., Yamamoto, T., Ogawa, K., Ohnishi, T., Daikuhara, Y., and Yamada, T. (2003) Increased hepatocyte growth factor level in cerebrospinal fluid in Alzheimer's disease. *Acta Neurol. Scand*. **107,** 81–86.
31. Takeuchi, D., Sato, N., Shimamura, M., Kurinami, H., Takeda, S., Shinohara, M., Suzuki, S., Kojima, M., Ogihara, T., and Morishita, R. (2008) Alleviation of Abeta-induced cognitive impairment by ultrasound-mediated gene transfer of HGF in a mouse model. *Gene Ther.* **15,** 561–571.
32. Kuwabara, Y., Ichiya, Y., Otsuka, M., Masuda, K., Ichimiya, A., and Fujishima, M. (1992) Cerebrovascular responsiveness to hypercapnia in Alzheimer's dementia and vascular dementia of the Binswanger type. *Stroke* **23,** 594–598.
33. Aslan, M. and Ozben, T. (2004) Reactive oxygen and nitrogen species in Alzheimer's disease. *Curr. Alzheimer Res*. **1,** 111–119.
34. Rowan, M.J., Klyubin, I., Cullen, W.K., and Anwyl, R. (2003) Synaptic plasticity in animal models of early Alzheimer's disease. *Philos. Trans. R. Soc. Lond. B Biol. Sci.* **358,** 821–828.
35. Lockhart, B.P., Rodriguez, M., Mourlevat, S., Peron, P., Catesson, S., Villain, N., Galizzi, J.P., Boutin, J.A., and Lestage, P. (2007) S18986: a positive modulator of AMPA-receptors enhances (S)-AMPA-mediated BDNF mRNA and protein expression in rat primary cortical neuronal cultures. *Eur. J. Pharmacol*. **561,** 23–31.
36. Destot-Wong, K.D., Liang, K., Gupta, S.K., Favrais, G., Schwendimann, L., Pansiot, J., Baud, O., Spedding, M., Lelièvre, V., Mani, S., and Gressens, P. (2009) The AMPA receptor positive allosteric modulator, S18986, is neuroprotective against neonatal excitotoxic and inflammatory brain damage through BDNF synthesis. *Neuropharmacology* **57,** 277–286.
